# Extracting Atomic Contributions to Binding Free Energy using Molecular Dynamics Simulations with Mixed Solvents (MDmix)

**DOI:** 10.2174/1570163819666211223162829

**Published:** 2022-03-28

**Authors:** Daniel Alvarez-Garcia, Peter Schmidtke, Elena Cubero, Xavier Barril

**Affiliations:** 1 Gain Therapeutics, Parc Cientific de Barcelona, Baldiri Reixac 10, 08029 Barcelona, Spain;; 2 Facultat de Farmacia, Universitat de Barcelona, Av. Joan XXIII 27-31, 08028 Barcelona, Spain;; 3 Catalan Institution for Research and Advanced Studies (ICREA), Passeig Lluis Companys 23, 08010 Barcelona, Spain

**Keywords:** Mixed solvents, MD simulations, structure-based drug discovery, binding free energy, atomic contribution, MDmix

## Abstract

**Background:**

Mixed solvents MD (MDmix) simulations have proved to be a useful and increasingly accepted technique with several applications in structure-based drug discovery. One of the assumptions behind the methodology is the transferability of free energy values from the simulated cosolvent molecules to larger drug-like molecules. However, the binding free energy maps (ΔG_bind_) calculated for the different moieties of the cosolvent molecules (e.g. a hydroxyl map for the ethanol) are largely influenced by the rest of the solvent molecule and do not reflect the intrinsic affinity of the moiety in question. As such, they are hardly transferable to different molecules.

**Method:**

To achieve transferable energies, we present here a method for decomposing the molecular binding free energy into accurate atomic contributions.

**Results:**

We demonstrate with two qualitative visual examples how the corrected energy maps better match known binding hotspots and how they can reveal hidden hotspots with actual drug design potential.

**Conclusion:**

Atomic decomposition of binding free energies derived from MDmix simulations provides transferable and quantitative binding free energy maps.

## 1 INTRODUCTION

Predicting the binding free energy (ΔG_bind_) of a drug candidate (ligand) to its pharmacological target (receptor) is the holy grail of structure-based drug design. The ΔG_bind_ of two molecules engaged in a non-covalent complex has a univocal relationship with the equilibrium constant (K_A_ = 1/K_D_ = exp(-ΔG_bind_/RT)), which is the experimental observable. Thus, computational chemistry methods strive to predict ΔG_bind_ in the hope that one day it will be possible to design drugs on the computer. Traditional methods, such as MM-PBSA [1] or the scoring functions implemented in docking software [2], aim to predict ΔG_bind_ from the protein-ligand interactions. However, such methods cannot properly take into account the effect of the solvent and the configurational diversity of the bound and unbound states (ensembles). As a result, they often deliver mediocre results. Molecular dynamics (MD) simulations, combined with the impressive software and hardware developments of the last decades, have opened the possibility of observing binding and unbinding events of a protein-ligand system and, thus, a direct route to the calculation of the binding constant [3]. When executed properly, this offers the most accurate results, well within the experimental error [4]. However, this is impractical for drug-like ligands because: i) many binding and unbinding events must be observed in order to attain statistically meaningful binding constants, and ii) potent ligands exhibit slow (dissociation) kinetics, resulting in binding half-lives longer than the simulation times currently amenable [5]. Solutions to this limitation have been proposed [6-8], but calculating ΔGbind for a single molecule of interest is a major effort even in the best case. In order to exploit this powerful approximation in a practical way, we and others proposed the use of MD simulations with mixed aqueous/organic solvents (MDmix) [9, 10] due to their small size, organic solvents display, fast diffusion, and binding rates. Furthermore, they can be simulated at relatively high concentrations (1% to 20%), which facilitates rapid convergence of the simulations. While the organic solvents *per se* are of no interest, the information they provide can be very useful. Indeed, experimentally it has been observed that organic solvents can be used to detect binding hotspots, thus pinpointing the functional sites of proteins [11, 12]. MDmix-type methods have become very popular, with an expanding number of applications that range from the original use in druggability prediction to receptor-based pharmacophore discovery, identification of displaceable water molecules, elucidation of cryptic pockets, or as a scoring function for docking (for an extensive review, see reference [13]). However, all these applications rely on a crucial assumption: the transferability of the ΔG_bind_ obtained for the organic solvents to larger ligands. Group transferability has a long tradition in drug design. As a notable example, the Free-Wilson analysis relies on this particular assumption to derive quantitative structure-activity relationships (QSAR) [14]. But the ΔG_bind_ values derived from MDmix simulations reflect the contribution of the entire solvent molecule. In order to obtain quantitative values, it is necessary to partition this magnitude into group contributions that are more accurate and transferable. In this article, we outline a rigorous partitioning method of our device [15], which we have been using very successfully for the discovery of allosteric pharmacological chaperones. First, we will outline the method. Then we will provide examples to illustrate its utility.

## 2 METHODS

The first step in MDmix is to prepare a system of interest (usually a biological macromolecule) for MD simulation in the usual way. Then the system is solvated using a pre-equilibrated solvent box containing a mixture of water and the organic solvent that will be used as a probe (*e.g*., ethanol). Next, a sufficiently long MD trajectory is produced. This generally involves multiple independent replicas that will be analysed together. In the final step, the space around the system is partitioned into volume elements (voxels), and the ΔG_bind_ of the solvent at each voxel is calculated from its observed density [16]. At the first order, the atomic contribution to ΔG_bind_ of atom *i* (ΔG^i^_bind_) is calculated using the expression:

ΔG^i^_bind_ = -k_B_T ln(N_i_/N_o_) (1)

where k_B_ is the Boltzmann constant, T is the temperature at which the simulation was run, N_i_ is the number of times that a particular group has fallen in a volume element, and N_o_ is the expected value if there were no contribution from the macromolecule. However, this assumes that each atom moves freely, which is clearly not the case for polyatomic molecules.

**Fig. (1):**
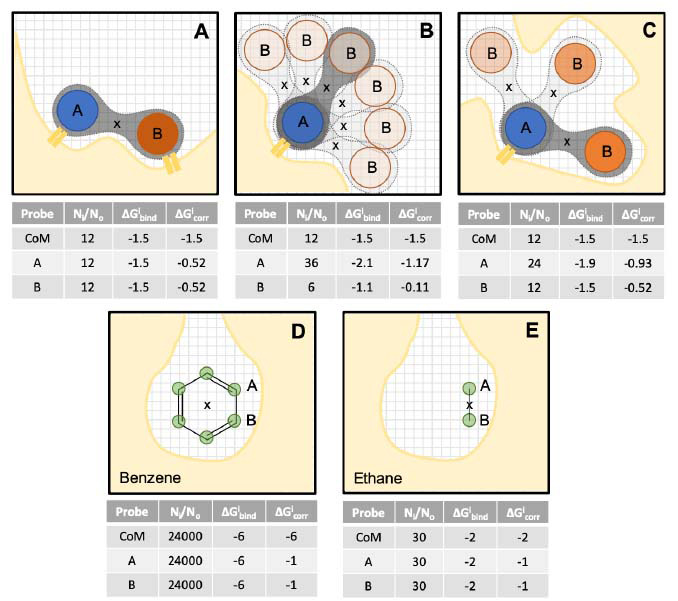
Calculation of atomic contributions to ΔG_bind_ on idealised systems. **Top:** Idealised 3-atom molecule bearing a hydrophobic head (atom A; blue; α^A^ = 0.35) and a polar tail (atom B; orange; α^B^ = 0.35). Total ΔG^M^_bind_ (as calculated from the density of the CoM (marked as **x**)) is the same in all cases, but they differ on the relative atomic densities. The tables show the raw (ΔG^i^_bind_) and corrected (ΔG^i^_corr_) atomic contributions for a particular binding mode of the molecule (dark-shaded background). **Bottom:** Size dependency of ΔG^i^_bind_ illustrated on an idealised site where benzene attains a total ΔG^M^_bind_ = 6 kcal/mol, and all atoms contribute equally. Two molecular probes of the same character but different sizes (benzene and ethane) afford very different ΔG^i^_bind_ but identical ΔG^i^_corr_ values. Protein surface is shown in ochre. The grid represents space discretization into volume elements

The MDmix method uses amphiphilic organic molecules to probe the interaction preferences of the biological macromolecule of interest. The use of amphiphilic molecules (*i.e*., those containing a polar head combined with a hydrophobic tail) provides two important advantages. On the one hand, due to their polar head, they are more soluble than purely hydrophobic solvents and can be simulated at higher concentrations (thus ensuring faster convergence) without the need for artificial potentials that prevent phase separation [10]. More importantly, the polar groups of organic molecules (*e.g*., a hydroxyl) behave quite differently than a purely polar solvent (*e.g*., water). Thus, the use of amphiphilic organic molecules is necessary to reliably identify and quantify polar interactions [17]. The downside to this choice is that binding to the surface of the macromolecule is the result of the interactions formed by each of the groups (hydrophobic and hydrophilic), which can be quite distinct. Fig. (**1**) (top) illustrates three ideal cases with the same total (molecule-based) ΔG_bind_, but disparate group contributions. In the first case (Fig. **1A**), both probe atoms make equally favourable contacts with the protein, and ΔG_bind_ is evenly distributed amongst them. In the second case (Fig. **1B**), only one group is making favourable contacts, while the other prefers to remain solvated, making no effective contribution to the total ΔG_bind_. The third case (Fig. **1C**) represents an intermediate situation, where one group makes the most important interaction, while the second group explores several energetically favourable positions. As shown, the group contributions (ΔG^i^_bind_) calculated using Equation 1 contain significant errors, because the observed densities for one atom are largely influenced by the interaction preferences of the other one (*i.e*., the assumption that the atoms move independently is not true). In order to obtain accurate and transferable group contributions, it becomes necessary to decouple the binding of the polar head from the binding of the hydrophobic tail. This can be done based on the relative densities of each group: those establishing stronger interactions will exhibit larger densities compared to the others. Fig. (**1**) (bottom) shows that atomic contributions (ΔG^i^_bind_) calculated in this way also have an artefactual dependency on the size of the molecule: irrespective of the chemical character of the atoms, bigger molecules can attain larger total ΔG_bind_ (absolute) values. This translates into larger observed densities of individual atoms, even if the atomic contributions (*i.e*., ligand efficiencies) are not better. The repartitioning scheme must be able to correct both of these situations, providing truly atom-specific contributions. As such, they should not surpass the experimentally-observed maximal atomic contributions, which rarely reach -1.0 kcal/mol and have a physical limit at -1.5 kcal/mol [18].

The decoupling procedure devised here is based on a comparison of the uncorrected atomic ΔG^i^_bind_ values with the expected value considering the ΔG_bind_ of the entire molecule (ΔG^M^_bind_). Firstly, we must calculate ΔG^i^_bind_ and ΔG^M^_bind_ using Equation 1. In the former case, we use the coordinates of the atomic nuclei to calculate densities and in the latter, the coordinates of the centre of mass (CoM) of the entire molecule. From ΔG^M^_bind_, we obtain the expected atomic contribution:

ΔG^i^_0_ = ΔG^M^_bind_ * α^i^ (2)

Where α^i^ is the fraction of ΔG^M^_bind_ contributed by atom *i* in the ideal situation where all atoms of the molecule contribute equally to the binding. For instance, in a molecule formed by N identical atoms (*e.g*., benzene, N=6), each atom would have an α value of 1/N. For molecules with non-identical atoms, several partitioning schemes are possible. For simplicity, let us assume that ΔG_bind_ is proportional to the solvent accessible surface area (SASA). Then, we can define α^i^ as the SASA fraction of atom *i* in molecule *M*:

α^i^ = SASA^i^ /SASA^M^ (3)

Note that, for flexible molecules, the SASA values should be averaged over all three-dimensional conformations (weighted by population). The corrected contribution of a particular atom can then be calculated as the uncorrected value minus the expected contribution of the rest of the molecule:

ΔG^i^_corr_ = ΔG^i^_bind_ – (ΔG^M^_bind_ – ΔG^i^_0_) (4)

Fig. (**1**) illustrates the impact of this correction in idealised systems. Since the CoM of the molecule can explore a range of positions when atom *i* localizes in a particular voxel, it is necessary to re-analyse the simulation, identifying the location of the CoM at each snapshot during the trajectory. The reported ΔG^i^_corr_ for a particular voxel is the average value across all snapshots in the simulation:

〈ΔG^i^_corr_〉 = å ΔG^i^_corr_ / N_i_ (5)

Where the summation runs from 1 to N_i_ (*i.e*., the number of times that atom *i* has fallen into that voxel). In the next section, we showcase two real case examples to demonstrate the power of the correction.

## 3 RESULTS

### 3.1 Example 1: Hsp90

Hsp90 is an oncology target that has become a testbed for structure-based drug design [19]. The primary interaction point in the active site of Hsp90, used by almost all known ligands, is the side-chain of Asp93, which acts as a powerful hydrogen bond acceptor [20]. We ran an MD simulation of the N-terminal domain of Hsp90 in apo form (initial structure 2XDK) [21] in a mixture of 20% v/v isopropanol/water mixture (equilibrated solvent box available for download at: http://mdmix.sourceforge.net). Then, the contributions to binding free energy were calculated using Eq. 1 on a cubic grid spanning the entire protein surface (grid spacing = 0.5Å in each dimension) for the methyl atoms (hydrophobic probe), the hydroxyl atom (polar probe), and the central atom (used as a proxy of the CoM). Fig. (**2**) shows the isocontour (surface encompassing voxels with equal values) of the hydrophobic probe at ΔG_bind_ = -1.5 kcal/mol (blue mesh). Note that this value is at the physical limit identified by Kuntz and Kollman [18], revealing that it is an overestimate caused by the use of a 4-atom molecule, as explained above. This visualization technique reveals five preferred binding sites (hot spots) for the hydrophobic probe. Four of them are in good agreement with the placement of hydrophobic moieties by known ligands. But the one closer to Asp93 overlaps with the preferred positions of polar atoms and can be attributed to the tight binding between the polar probe and Asp93 (similar to the situation described in Fig. **1B**). After correction (red surface isocontour), this presumed binding hot spot disappears, confirming that it was largely caused by the interaction preferences of the polar head rather than intrinsic interaction preferences of the hydrophobic tails. Interestingly, the same correction expands the hydrophobic hot spot at the bottom of the image, while the other three sites are not significantly affected by the correction. Note that the values depicted in this case (ΔGcorr = -0.7 kcal/mol) are in much better agreement with the expected atomic contribution for real ligands [18]. Thus, we conclude that the correction works as expected, eliminating artefacts due to the interdependence of the various atoms in a solvent molecule used as a probe and removing the size-dependency of the uncorrected atomic contributions. In consequence, the corrected free energy grids are much better suited to guide the rational design of ligands to macromolecular targets, particularly when there is no previous information about ligands.

### 3.2 Example 2: Allosteric Site of GA1

Glutaric acidemia type I (also called glutaric aciduria type I, GA1) is a rare but serious inherited disorder in which the body is unable to process certain amino acids properly.

GA1 patients have inadequate levels of the mitochondrial enzyme glutaryl-CoA dehydrogenase (GCDH), which helps break down the amino acids lysine, hydroxylysine, and tryptophan. Excessive levels of these amino acids and their intermediate breakdown products in the blood, urine, and tissues can be toxic, causing severe health problems [22, 23]. The clinical course of GA1 often features an episode of acute metabolic encephalopathy, which results in irreversible striatal injury. A burdensome dietary and pharmacological treatment does not prevent devastating neurological complications in at least 15% of the patients [22, 23]. GCDH deficiency often occurs due to mutation-induced protein misfolding [24]. Therefore, the rescue of the misfolded proteins by pharmacological chaperones is a promising novel therapeutic approach for GA1.

The structure of Human Glutaryl-CoA Dehydrogenase in complex with the cofactor FAD (PDB code 1SIQ) was used as a starting point for the simulation to generate the biological (homotetramer) from the single-chain found in the asymmetric unit. FAD was kept in the MD simulation because the interest was to find allosteric ligands that would not compete with the substrate or the cofactor. In this case, we used ethanol as probe solvent, using a pre-equilibrated box of ethanol/water at 20% (v/v), as described [17]. As shown in Fig. (**3**), a preferred binding site for ethanol was found near Thr65, but originally it appeared too small to offer significant binding opportunities for a drug-like ligand. After subsequent correction of the free energy values with the above-described procedure, three additional hydrophobic hot spots emerged. They were largely caused by a conformational change of the side-chains of Lys313 and Asn248, which opened up a large hydrophobic patch. This illustrates another advantage of MDmix as a binding-site mapper: as the protein is allowed a significant amount of conformational flexibility [16], the presence of hydrophobic probe solvents facilitates the opening of cryptic pockets [25-29]. As the polar head of ethanol shows a scarce affinity for the emerged hydrophobic patch, in the absence of the correction, the apparent affinity of the methyl group was underestimated. The centre of four binding hot spots was used to define pharmacophoric points (1 hydrogen bond acceptor, plus three hydrophobic groups) that were used as restraints in a docking-based virtual screening with the rDock program [30].

## CONCLUSION

Mixed solvent techniques have become quite popular [13, 31]. However, there is no consensus yet on the conditions that should be used, including the type of solvents, concentrations, use of solvent-solvent repulsive potentials, protein conformational restraints, length of simulations, etcetera. Furthermore, the technique is fundamentally used in a qualitative way. This may be sufficient to elucidate binding sites (the main use reported so far) but falls short for more quantitative application. Here we have presented a method to decompose the binding free energy (a molecular property) into atomic contributions that are more accurate and transferable to larger ligands. An alternative approach to ensure transferability is to simulate a very large set of solvent probes, each representing a chemical moiety present in typical drugs. This was recently demonstrated by Yanagisawa and co-workers using a set of 138 cosolvents [32]. Simulation of a much smaller set of cosolvents containing the essential atom types, followed by atomic partitioning of ΔG_bind,_ is far more efficient [33, 34]. When using non-corrected atomic contributions, cosolvent-based simulations can yield relative binding affinities as accurate as the free energy perturbation (FEP) methods, which are considered the gold standard in structure-based drug design [35]. The atomic repartition scheme presented here should bring about a further increase in accuracy [15]. Besides the theoretical background of the partitioning scheme, here, we have illustrated the improvement in the predictions with two visual examples. Future articles will disclose practical applications of the method and investigate the use of said atomic contributions to predict (relative) binding free energies on congeneric series of ligands.

## Figures and Tables

**Fig. (2):**
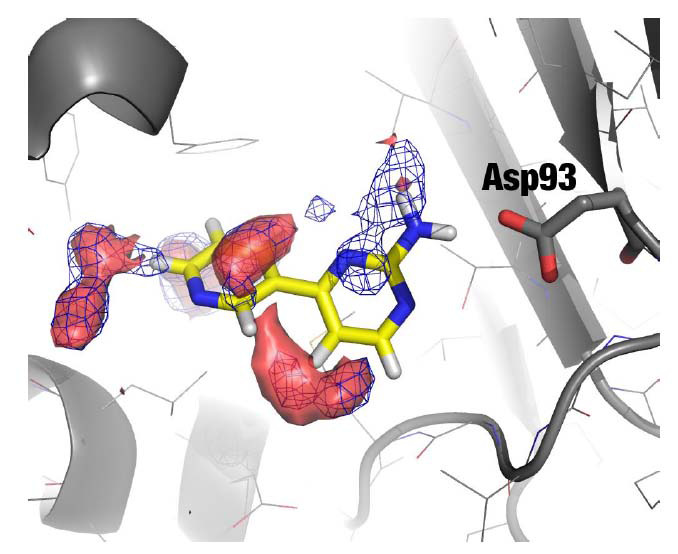
Contour plots showing the optimal interaction sites of a hydrophobic probe (methyl of isopropanol) in an uncorrected grid (blue mesh; ΔG^i^_bind_ = -1.5 kcal/mol) and after correction (red transparent surface; ΔG^i^_corr_ = -0.7 kcal/mol). The ligand (yellow sticks; PDB code 2XDK) is displayed for reference purposes only. The protein is displayed in the grey cartoon (backbone) and lines (atoms), except for Asp93, shown in the sticks

**Fig. (3):**
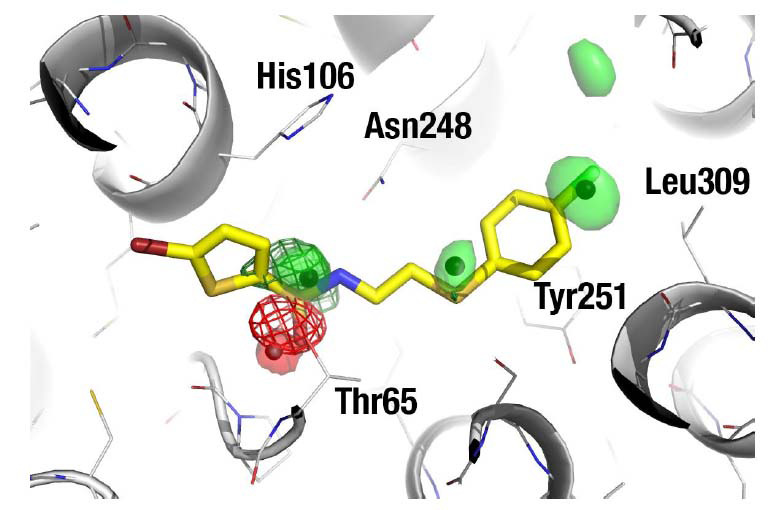
Contour plots showing the optimal interaction sites of a hydrophobic probe (methyl of ethanol; green mesh) and a polar probe (hydroxyl of ethanol; red mesh), both at ΔG_bind_ = -1.5 kcal/mol. They identify a preferred binding site for ethanol next to Thr65. However, the site appears to lack any additional binding hot spot, suggesting that it is too small to be druggable. After correction, the hot spots are maintained (red and green transparent surfaces; ΔG_corr_ = -1.0 kcal/mol), but three additional hydrophobic hot spots emerge, revealing that the binding site offers substantial binding opportunities to a drug-like ligand. The centre of the corrected hot spots used to define a pharmacophore are shown as black dots. The ligand shown in yellow is an example virtual screening hit

## Data Availability

The data supporting the findings of the article is available within the article.
